# Rapid Determination of Palmitic Acid Content in Edible Oils Using Vis-NIR Reflectance Spectroscopy and Deep Learning Models

**DOI:** 10.3390/foods15111888

**Published:** 2026-05-27

**Authors:** Ning Su, Huiliang Yang, Qiyun Zheng, Fei Lin, Taosheng Xu

**Affiliations:** 1 School of Artificial Intelligence and Big Data, Hefei University, Hefei 230601, China; oksuning@mail.ustc.edu.cn; 2 Internet College, Anhui University, Hefei 230036, China; y23111001@stu.ahu.edu.cn; 3 Institute of Intelligent Machines, Hefei Institutes of Physical Science, Chinese Academy of Sciences, Hefei 230031, China; qy_zheng@stu.ahau.edu.cn (Q.Z.); feilin@iim.ac.cn (F.L.)

**Keywords:** edible oils, palmitic acid, Vis-NIR reflectance spectroscopy, CARS, deep learning, ResNet

## Abstract

Fatty acid abundance is a key parameter for evaluating the quality of edible oils. This study developed a rapid and non-destructive method for predicting palmitic acid content in edible oils by combining visible-near-infrared (Vis-NIR) reflectance spectroscopy with deep learning models. A total of 1740 reflectance spectra in the range of 350–2500 nm were collected from 87 brands of edible oils, including peanut, soybean, corn, sunflower, rapeseed, sesame, and olive oils. Reference values of palmitic acid content were determined via gas chromatography–mass spectrometry (GC-MS). Two conventional machine learning models (SVR and KNN) and four deep learning models (1D-CNN, 1D-ResNet, 1D-Inception, and 1D-Inception-ResNet) were developed and compared using both full-spectrum data and CARS selected characteristic wavelengths. Among the full-spectrum models, the designed 1D-ResNet model achieved the best performance, with the determination coefficient of prediction ($R_{p}^{2}$) of 0.9027 and the root mean square error of prediction ($RMSE_{p}$) of 1.13 in the prediction dataset. The proposed 1D-Inception-ResNet model yielded the best prediction results based on the 91 selected informative wavelengths via competitive adaptive reweighted sampling (CARS), achieving an $R_{p}^{2}$ of 0.9825 and an $RMSE_{p}$ of 0.4804 in the prediction dataset. The experimental results indicated that Vis-NIR reflectance spectroscopy combined with informative wavelength selection and deep learning models provided an effective strategy for rapid prediction of palmitic acid content in edible oils.

## 1. Introduction

Edible vegetable oils are essential components of the human diet and major sources of dietary lipids [1,2]. They contain abundant fatty acids and fat-soluble vitamins that are closely associated with human health [3,4]. The composition and relative proportions of fatty acids in vegetable oils are therefore important indicators of nutritional value and quality [5]. Accurate determination of fatty acid composition is also important for adulteration detection [6], quality evaluation, and origin tracing [7]. Among the different fatty acids present in edible oils, palmitic acid is an important saturated fatty acid whose content contributes to the nutritional and compositional characterization of oil products.

At present, fatty acid analysis in edible oils mainly relies on chromatography, spectroscopy, nuclear magnetic resonance (NMR), and electronic nose technologies. Gas chromatography–mass spectrometry (GC-MS) is widely used for quantitative fatty acid analysis because of its high accuracy and sensitivity, with reported relative standard deviations below 9% [8]. Other spectroscopic methods have also been explored. For example, Dogruer et al. used fluorescence spectroscopy and mid-infrared spectroscopy to quantify major fatty acids and free fatty acids in sunflower oil blends containing pumpkin seed, grape seed, black cumin, and sesame oils [9]. However, chromatography and NMR generally require laborious sample preparation, expensive instrumentation, and relatively long analysis times, which limit their suitability for rapid or on-site applications. Electronic nose systems also suffer from sensor aging and insufficient analytical accuracy in some applications [10].

Spectroscopic techniques provide non-destructive and efficient alternatives for food analysis. Commonly used methods include Raman spectroscopy, fluorescence spectroscopy, and near-infrared (NIR) spectroscopy. Temiz et al. applied Raman spectroscopy to cold-pressed oil adulteration analysis and reported cross-validation errors ranging from 0.05 to 0.25 depending on the oil type [11]. Poulli et al. used fluorescence spectroscopy to detect sunflower oil adulteration in olive oil, achieving a detection sensitivity of 3.4% [12]. Nevertheless, Raman spectroscopy usually depends on specific excitation sources and may suffer from relatively low sensitivity. Fluorescence spectroscopy generally requires more specialized instrumentation and is more sensitive to environmental and sample matrix interferences. By contrast, NIR spectroscopy has been increasingly applied in food analysis because organic molecules containing hydrogen-bearing groups exhibit overtone and combination-band absorption in the NIR region [13,14]. As a result, NIR spectra contain rich chemical information related to molecular structure and composition [15,16]. The combination of visible and NIR spectral regions further increases the amount of useful information available for analysis. Vis-NIR reflectance spectroscopy therefore offers a broad spectral range, rapid measurement, simple operation, and strong potential for portable qualitative and quantitative applications.

To exploit these advantages, this study used Vis-NIR reflectance spectroscopy to predict palmitic acid content in edible oils. The relationship between spectral variables and chemically measured reference values was modeled using data-driven methods. Support vector regression (SVR) [17] and k-nearest neighbor (KNN) [18] are conventional machine learning methods that have been used for NIR spectral analysis, including the identification of edible oils with different storage periods [19]. However, traditional machine learning algorithms often rely heavily on handcrafted features and may show limited generalization when spectral information is highly redundant, nonlinear, and structurally complex. Deep learning provides a more powerful alternative because it can automatically learn representative features directly from raw spectral inputs. Among deep learning architectures, convolutional neural networks (CNNs) are well suited for spectral sequence modeling because they can extract local and hierarchical features along the wavelength axis. However, increasing network depth in conventional CNNs may lead to optimization difficulties such as gradient vanishing and performance degradation. Residual Network (ResNet) addresses this issue by introducing shortcut connections that facilitate information propagation in deep architectures [20]. Inception further improves computational efficiency by employing multi-branch convolutions to extract features at multiple receptive-field scales while controlling the number of parameters [21]. Although ResNet-based and Inception-based architectures have been widely studied in image analysis, their application to Vis-NIR spectral modeling remains relatively limited. Accordingly, this study investigated the feasibility of combining Vis-NIR reflectance spectroscopy with machine learning and deep learning for quantitative prediction of palmitic acid content in edible oils. Two conventional machine learning models, namely, SVR and KNN, and four deep learning models, namely, 1D-CNN, 1D-ResNet, 1D-Inception, and 1D-Inception-ResNet, were developed and compared. In addition, competitive adaptive reweighted sampling (CARS) was used to select informative wavelengths and construct simplified prediction models. The performance was further compared with that of the corresponding full-spectrum models.

## 2. Materials and Methods

### 2.1. The Oil Samples

A total of 87 commercial edible vegetable oil brands were randomly collected in this study. The samples were purchased from Walmart stores in Hefei, China, and from JD.com (an online retailer). The collection comprised 15 peanut oil brands, 15 soybean oil brands, 11 corn oil brands, 11 sunflower oil brands, 13 rapeseed oil brands, 9 sesame oil brands, and 13 olive oil brands. All of the edible oil samples were firstly stirred evenly to ensure homogeneity. For each brand, eight replicate samples (20 mL each) were taken for subsequent analysis. Therefore, a total of 696 oil samples (87 brands × 8 replicates) were collected in our experiment. Among them, four replicates per brand were used for gas chromatography–mass spectrometry (GC–MS) to determine the palmitic acid content. The remaining 348 samples (87 brands × 4 replicates) were used for Vis-NIR reflectance spectrum acquisition.

### 2.2. Vis-NIR Reflectance Spectrum Acquisition

**Figure 1:**
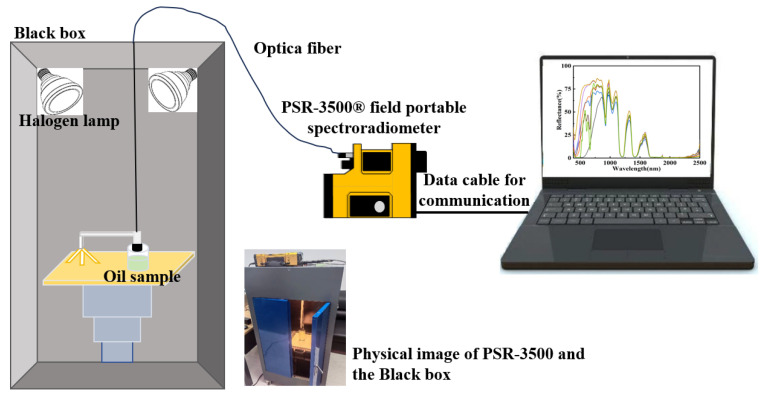
The Vis-NIR reflectance spectral acquisition system. The color lines are examples of Vis-NIR reflectance spectroscopy.

To acquire a reliable Vis-NIR spectrum from the edible oil samples, a dedicated reflectance spectral acquisition system was designed and constructed in our experiment, as shown in Figure 1. The reflectance spectral acquisition system consisted of two main components. The key component for acquiring Vis-NIR reflectance spectra of oil samples was a PSR-3500® field portable spectroradiometer (Spectral Evolution, Lawrence, MA, USA), which was equipped with three array detectors: one 512-element silicon (Si) detector and two 256-element indium gallium arsenide (InGaAs) array detectors. The wavelength range of the Vis-NIR reflectance spectrum for edible oil samples was 350–2500 nm. The second component was a computer installed with DARWin^TM^ SP Data Acquisition Software, Version 1.2 (Spectral Evolution, Lawrence, MA, USA) for data collection and processing. Reflectance measurements were highly susceptible to environmental factors such as illumination and structural variations in the measurement setup. Therefore, we designed a custom-built reflectance measurement platform with a dark box to ensure consistent structured optical conditions. In the dark box, the hyperspectral sensor probe was connected to the spectroradiometer body via an optical fiber, and a halogen light source, a tripod, a polyvinyl chloride (PVC) white reference board, and a sample stage were installed for Vis-NIR reflectance spectrum measurement. Therefore, all spectral acquisitions were conducted inside a dark box to minimize external light interference. Prior to measurement, the halogen lamp was preheated for 30 min to ensure a stable light output. Meanwhile, a cooling fan installed in the dark box was activated to prevent excessive temperature rise during operation. Before data collection, the instrument was calibrated according to the instructions of the spectroradiometer. A 25 mL transparent glass beaker was placed at the center of the sample stage, with a PVC white board positioned at the bottom of the beaker to serve as a reflectance reference background. The fiber-optic probe, fixed at a field-of-view (FOV) angle of 8°, was mounted vertically above the beaker using a tripod to maintain consistent measurement geometry. A working distance of 3 cm was maintained between the spectral acquisition lens and the surface oil samples. For each edible oil brand, four replicates of 20 mL each were prepared for spectral acquisition. Each replicate was transferred into a separate 25 mL glass beaker to ensure independent measurement. This replicate strategy allowed us to account for potential within-brand variability and to obtain representative reflectance spectra. In practice, edible oils may contain colloidally suspended particles, which can contribute to scattered radiation in addition to surface reflection. To minimize such effects, five spectra were collected from different positions (center, upper, lower, left, and right regions of the liquid surface) for each replicate. This multi-point sampling strategy effectively reduces scattering influence. Each spectrum was acquired within approximately 2–4 s. In total, 1740 reflectance spectra of the 348 oil samples in 87 brands were collected for data analysis.

### 2.3. Content Determination of Palmitic Acid in Edible Oils Using GC-MS

#### 2.3.1. Experimental Configuration of GC-MS

The palmitic acid content in the edible oil samples was measured via GC-MS and used as the reference value for model training and data analysis. In our study, the content determination analysis of palmitic acid was performed using GCMS-QP2010 SE (Shimadzu Corporation, Kyoto, Japan). The chemical reagents used for GC–MS analysis, including n-hexane, methanol, hydrochloric acid, and potassium hydroxide, were of analytical grade and obtained from Sinopharm Chemical Reagent Co., Ltd. (Shanghai, China). Palmitic acid methyl ester (purity ≥ 99.0%) was purchased from Aladdin Biochemical Technology Co., Ltd. (Shanghai, China) and used as a reference standard. High-purity helium (99.99%) used as the carrier gas in GC–MS analysis was supplied by Hefei Zhongyi Chemical Products Co., Ltd. (Hefei, China). An analytical balance, ultrasonic cleaner, and centrifuge were used for sample preparation. Chromatographic separation was achieved using a DB-5MS fused-silica capillary column (30 m length × 0.25 mm inner diameter, film thickness 0.25 μm). The high-purity helium was employed at a constant flow rate of 1.0 mL/min. The oven temperature program was set as follows: initial temperature of 60 °C, increased to 215 °C at a rate of 15 °C/min, then to 250 °C at 10 °C/min, followed by heating to 260 °C at 2 °C/min, and finally to 280 °C at 5 °C/min. The injector temperature was maintained at 250 °C. Samples (1 μL per injection) were introduced in split mode with a split ratio of 40:1. Mass spectrometric detection was carried out using an electron ionization (EI) source operating at 70 eV. The ion source temperature was set at 250 °C. The electron multiplier voltage was 1.5 kV. The solvent delay time was 10 min, and mass spectra were acquired at a rate of 20 spectra per second.

#### 2.3.2. Measurement Method of Palmitic Acid

Palmitic acid is a long-chain fatty acid with low volatility and a relatively high boiling point. As a result, direct injection into the GC-MS system is generally unsuitable, because incomplete vaporization can lead to poor chromatographic separation and unsatisfactory peak shapes. Fatty acid methyl ester (FAME) derivatization improves volatility and promotes efficient chromatographic separation, allowing individual fatty acids to be detected as well-resolved peaks. Therefore, palmitic acid was converted into its corresponding methyl ester prior to analysis in our experiments. The edible oil samples were subjected to methyl esterification. Briefly, 100 mg of oil sample was introduced into a 10 mL screw-cap test tube, and 2 mL of n-hexane was then added. The mixture solution was ultrasonically treated for 10 min to ensure complete dissolution. Subsequently, 2 mL of 0.5 mol/L potassium hydroxide in methanol solution was added, and the mixture was ultrasonicated for 5 min to simultaneously achieve saponification and methyl esterification. After the reaction, 2 mL of hydrochloric acid was added to neutralize excess potassium hydroxide. The mixture was then incubated in a thermostatic water bath at 35 °C for 3 min. The upper organic phase-containing fatty acid methyl ester was collected and filtered through a 0.45 μm membrane filter into a GC vial. The filtered solution was subsequently analyzed via GC–MS to determine the palmitic acid content in the edible oil samples.

### 2.4. Selection of Characteristic Wavelengths of Vis-NIR Reflectance Spectra

CARS is a variable selection method that integrates Monte Carlo sampling with partial least squares regression (PLSR). First, Monte Carlo sampling is performed by randomly selecting a subset of samples from the calibration set to construct a PLSR model in each sampling run. The absolute values of the regression coefficients obtained from the PLSR model are used as an importance index for variable evaluation. Variables with relatively small absolute regression coefficients are gradually eliminated, while those with larger coefficients are retained. The number of variables to be removed in each iteration is determined by an exponentially decreasing function (EDF), which enforces a progressive reduction in variables. Subsequently, adaptive reweighted sampling (ARS) is applied to further eliminate less informative variables through a competitive selection mechanism. For each variable subset generated during the sampling process, a PLSR model is established, and the root mean square error of cross-validation (RMSECV) is calculated. The subset corresponding to the minimum RMSECV is considered optimal, and the associated wavelengths are selected as the characteristic variables. In summary, each sampling cycle of the CARS algorithm consists of four sequential steps: (1) Monte Carlo sampling, (2) EDF-based forced variable elimination, (3) ARS-based competitive variable selection, and (4) RMSECV evaluation of variable subsets. In this study, the CARS was employed for characteristic wavelength selection of the Vis-NIR reflectance spectra.

### 2.5. Model Establishment for Palmitic Acid Content Prediction

To predict palmitic acid content in edible oil samples from Vis-NIR reflectance spectra, two conventional machine learning models, namely, SVR and KNN regression, as well as four deep learning architectures, were developed. For the deep learning models, a one-dimensional convolutional neural network (1D-CNN) architecture was employed because each sample was represented by a one-dimensional spectral sequence ordered by wavelength. Compared with the standard two-dimensional CNN (kernel size: n×n), 1D-CNNs are more suitable for spectral sequence analysis because they can efficiently capture local dependencies, absorption patterns, and hierarchical features along the spectral axis while preserving the intrinsic sequential structure of the data [22,23]. It is worth noting that SVR and KNN directly employed conventional machine learning methods, whereas the four deep-learning models were newly developed in this study for Vis-NIR reflectance spectra analysis.

#### 2.5.1. Support Vector Regression

SVR is a supervised learning method that performs nonlinear regression through kernel-based mapping in the feature space and is suitable for small-sample datasets. The predictive performance of SVR is strongly influenced by the penalty parameter, which controls the trade-off between model complexity and prediction error. An excessively large penalty parameter may lead to overfitting, whereas an excessively small penalty parameter may result in underfitting. In this study, grid search was used to optimize the SVR hyperparameters.

#### 2.5.2. K-Nearest Neighbor Regression

KNN regression predicts continuous responses according to the values of neighboring samples in the feature space. In general, the predicted value is obtained from the mean of the *K* nearest neighbors or from a distance-weighted mean. The number of nearest neighbors, *K*, is a key parameter affecting model performance. A small *K* may increase the risk of overfitting, whereas a large *K* may oversimplify the model and lead to underfitting.

#### 2.5.3. Development of 1D-CNN Architecture for Palmitic Acid Content Prediction

**Figure 2:**
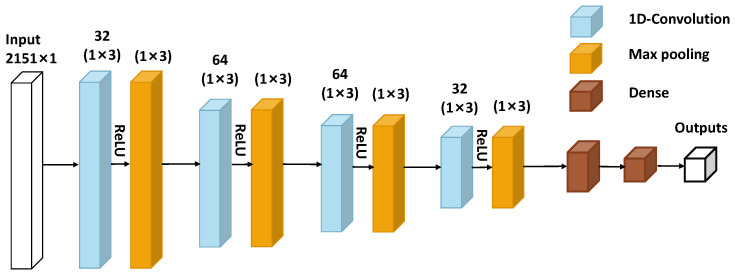
The 1D-CNN architecture for palmitic acid prediction.

A 1D-CNN architecture was developed to quantitatively predict palmitic acid content from Vis-NIR reflectance spectra. As illustrated in Figure 2, the proposed network comprised four consecutive one-dimensional convolutional blocks, each followed by a nonlinear activation layer (ReLU) and a max-pooling operation for progressive feature extraction and dimensionality reduction. Through these stacked convolution stages, local spectral patterns at different abstraction levels were captured and transformed into compact feature representations. The final feature maps were then flattened and passed through two fully connected (dense) layers, and a single-node output layer was used to generate the predicted palmitic acid content.

#### 2.5.4. Development of 1D-ResNet Architecture for Palmitic Acid Content Prediction

**Figure 3:**
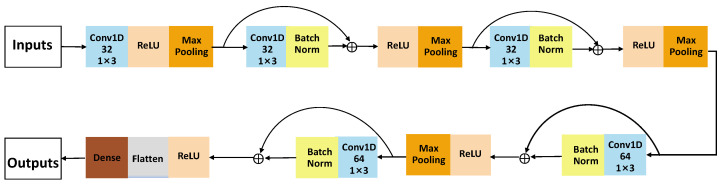
Structure of 1D-ResNet for palmitic acid prediction.

In this study, a 1D-ResNet model was constructed for the quantitative prediction of palmitic acid content from Vis-NIR spectral data. As shown in Figure 3, the proposed 1D-ResNet model for spectral regression started with a one-dimensional convolution (Conv1D) layer with 32 filters and a kernel size of 3, followed by a ReLU activation layer and a max-pooling layer. The extracted features were then passed through four residual blocks. The first two residual blocks used Conv1D layers with 32 filters, whereas the latter two employed 64 filters to further enhance feature representation. In each residual block, the convolutional output was batch-normalized and then fused with the corresponding shortcut connection through element-wise addition. Finally, the learned feature maps were flattened and fed into a fully connected layer to output the predicted palmitic acid content.

#### 2.5.5. Development of 1D-Inception Network for Palmitic Acid Content Prediction

**Figure 4:**
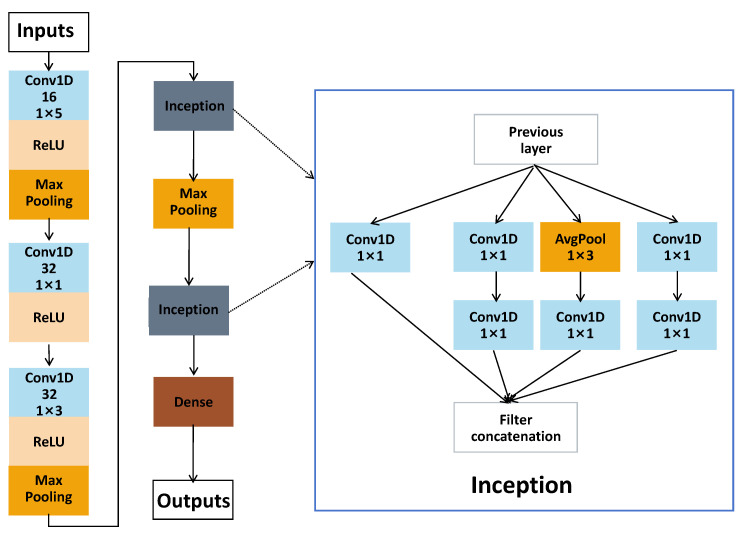
Structure of 1D-Inception Network for palmitic acid prediction.

The Inception network was composed of multiple Inception modules arranged in series and was designed to extract features at multiple scales [21]. In each Inception module, parallel branches with different convolutional kernel sizes were used to capture local patterns of different receptive field widths. The Inception architecture designed in this study is shown in Figure 4. The model consisted of two cascaded Inception modules to capture spectral features at multiple scales. In each module, the input was divided into parallel branches comprising convolutional layers with kernel sizes of 1, 3, and 5, together with an average-pooling branch with a kernel size of 3. The outputs of these branches were concatenated and forwarded to the next layer. To reduce computational cost, a convolution with a kernel size of 1 was applied before the branches with kernel sizes of 3 and 5 for dimensionality reduction. The final feature maps were then passed through a fully connected layer to output the predicted palmitic acid content.

#### 2.5.6. Development of 1D-Inception-ResNet Network for Palmitic Acid Content Prediction

**Figure 5:**
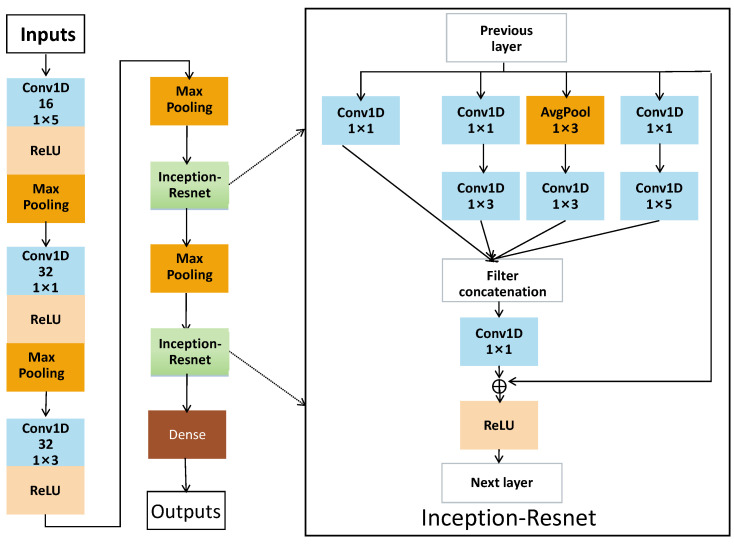
Structure of 1D-Inception-ResNet Network for palmitic acid prediction.

The Inception-ResNet network integrated Inception modules with residual connections, allowing the model to capture multi-scale features while facilitating the training of deep networks. By introducing shortcut connections into the Inception module, the input can be directly added to the transformed output, thereby enhancing information flow across layers and alleviating gradient vanishing during training. As shown in Figure 5, the proposed 1D-Inception-ResNet model was constructed by embedding residual connections into the original Inception architecture. After each Inception module, a projection layer was employed to adjust feature dimensions and match the input and output representations for residual addition. This design allowed the network to effectively integrate multi-scale spectral features and residual mappings.

### 2.6. Model Evaluation Metrics

To evaluate the performance of the proposed models, the dataset was divided at the sample level. Specifically, the 87 edible oil brands comprised 348 independent samples. These 348 samples were split into a calibration set and a prediction set at a ratio of 2:1 at the brand level, resulting in 1160 spectra from 58 brands in the calibration set and 580 spectra from the remaining 29 brands in the prediction set. It should be noted that the allocation of brands to the calibration set and prediction set also approximately adhered to the 2:1 ratio within each edible oil type. The coefficient of determination ($R^{2}$) and the root mean square error (RMSE) were used to assess the goodness of fit and predictive performance of the prediction models. The metrics were calculated as follows:

(1) $$R^{2}=1-\frac{RSS}{TSS}$$

(2) $$RMSE=\sqrt{\frac{1}{n}\sum_{i=1}^{n}{\left(y_{i}-{\hat{y}}_{i}\right)}^{2}}$$

where $RSS=\sum_{i=1}^{n}{\left(y_{i}-{\hat{y}}_{i}\right)}^{2}$ and $TSS=\sum_{i=1}^{n}{\left(y_{i}-\bar{y}\right)}^{2}$, *n* is the number of samples, *i* denotes the *i*-th sample, $y_{i}$ is the reference value of the sample, ${\hat{y}}_{i}$ is the predicted value for the samples, and $\bar{y}$ is the mean of the reference values. The coefficient of determination ($R^{2}$) reflects the goodness of fit of a regression model. A value closer to 1 indicates better model performance. For the calibration set and prediction set, the coefficient of determination is denoted as $R_{c}^{2}$ and $R_{p}^{2}$, respectively. Meanwhile, RMSE measures the deviation between the predicted values and the reference values. A smaller RMSE indicates higher prediction accuracy. The RMSE for the calibration set and prediction set are denoted as $RMSE_{c}$ and $RMSE_{p}$, respectively. In general, closer agreement between calibration and prediction results indicates better robustness and generalization ability of the model.

## 3. Results

### 3.1. Spectral Characteristics of the Edible Oil Samples

**Figure 6:**
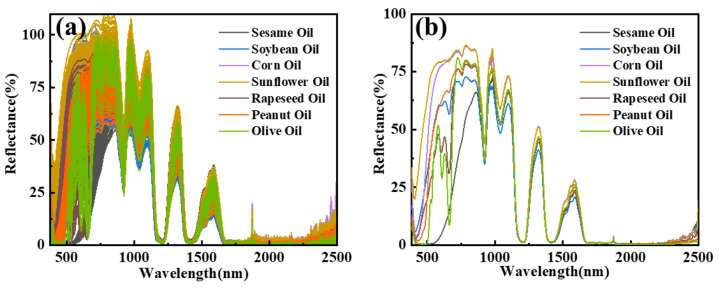
The reflectance spectra of the 87 edible oils. (**a**) Original reflectance spectra of all oil samples. (**b**) The mean reflectance spectra of seven oil samples.

In this study, a total of 1740 Vis-NIR reflectance spectra covering the wavelength range of 350–2500 nm were obtained from 87 brands of edible oils. For each brand, four replicate samples were prepared, and five spectra were measured for each sample. Figure 6a,b show the raw reflectance spectra of all edible oil samples and the mean reflectance spectra of each oil type, respectively. The mean reflectance spectra were obtained by averaging all spectra within each of the seven edible oil types. Further analysis of the spectral characteristics of the seven edible oil types was conducted based on the mean spectra.

As shown in Figure 6b, in the range of 850–2500 nm, all oil types exhibited a similar overall spectral trend, with differences mainly observed in reflectance intensity. The spectral features of edible oils in this region were primarily associated with strong molecular vibrations of functional groups such as C-H, O-H, and N-H [24]. The peaks at 856 nm, 1098 nm, and 1320 nm were related to C-H stretching vibrations, whereas the peaks near 980 nm and 1586 nm were attributed to O-H bending vibration and the second overtone of N-H, respectively [25,26]. In contrast, distinct differences among oil types were observed in the visible-to-short-wave near-infrared region (350–850 nm). The reflectance spectrum of sesame oil showed a continuously increasing trend across this range. The corn oil and sunflower oil exhibited a rapid increase followed by a more gradual change, and both showed the highest reflectance intensity among all oil samples. In addition, the corn oil and sunflower oil oils displayed a trough around 760 nm and a peak near 790 nm, while sunflower oil also showed a slight trough near 660 nm. The spectral profile of peanut oil was similar to that of sunflower oil, although its reflectance intensity was lower throughout this region. Except for sesame oil, soybean oil showed the weakest signal in the range of 700–900 nm and exhibited a broad peak near 620 nm. The spectral variation of olive oil was similar to that of rapeseed oil. However, olive oil showed a weak peak near 520 nm and a larger variation amplitude than rapeseed oil. The spectral differences observed in the 350–850 nm range were mainly attributed to chlorophyll and carotenoid pigments present in edible oils [27]. These spectral differences provided preliminary evidence for the feasibility of using reflectance spectroscopy to analyze palmitic acid in edible vegetable oils. However, due to the substantial overlap present among the reflectance spectra of different oil samples, it was difficult to quantify these subtle differences based solely on visual inspection of the spectra. Therefore, it was necessary to combine characteristic wavelength selection with machine learning and deep learning methods to fully exploit the spectral information and enable quantitative prediction of palmitic acid content in edible oils.

### 3.2. Palmitic Acid Quantification via GC-MS

**Figure 7:**
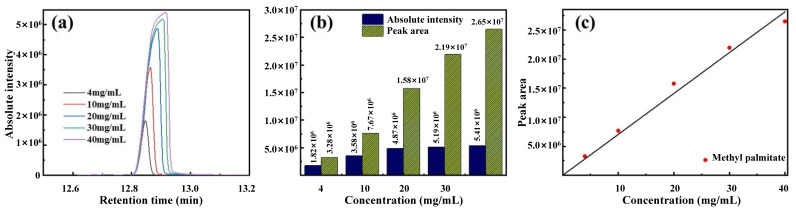
Calibration results for palmitic acid methyl ester obtained via GC-MS. (**a**) Retention time and absolute intensity of methyl palmitate at different concentrations, (**b**) absolute intensity and peak area of methyl palmitate at different concentrations, and (**c**) the fitting relationship between concentration of methyl palmitate and peak area.

For the quantification of palmitic acid in GC-MS, a calibration curve was established using commercially purchased palmitic acid methyl ester (purity ≥ 99.0%). The standard compound was dissolved in n-hexane to prepare a series of solutions with mass concentrations of 4, 10, 20, 30, and 40 mg/mL. For each concentration, 0.5 mL of the prepared solution was transferred into a GC vial and analyzed via GC–MS under the operating conditions described in Section 2.3.1. The chromatographic results are presented in Figure 7. Figure 7a shows the retention time, absolute intensity, and peak area of palmitic acid methyl ester at the five different concentrations. Figure 7b illustrates the relationship between absolute intensity and peak area at varying concentrations. A linear regression model was constructed using the least squares method to describe the relationship between palmitic acid concentration (*x*) and peak area (*y*). The resulting calibration equation was y=703,593x. The $R^{2}$ of the calibration equation was 0.99421. As shown in Figure 7c, the calibration curve showed good linearity within the tested concentration range. For quantitative analysis of the 87 commercial edible vegetable oil brands, four independent subsamples were prepared for each brand and subjected to the methyl esterification procedure described above, followed by GC–MS analysis. The mean value of the four measurements was used as the reference palmitic acid content for each oil brand.

**Figure 8:**
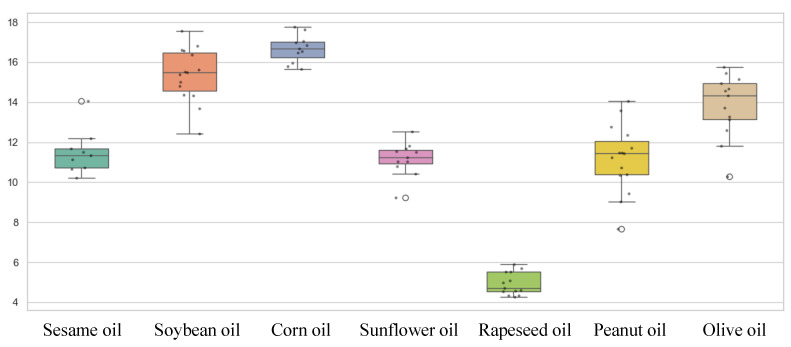
The box-plot of the palmitic acid content in 87 brands of edible oils (Unit: %). Some of the dots (hollow circle) that exceed the top or bottom of the box are the outliers.

As shown in Figure 8, the box-plot of the palmitic acid content in 87 brands of edible oils revealed variability across different oil types. The overall content ranged from approximately 4% to 18%. Corn oil displayed the highest median palmitic acid content among the tested oil types, with a mean of 16.67% and a standard deviation (SD) of 0.68. Soybean oil also showed a considerable palmitic acid content (mean = 15.38%, SD = 1.34). However, the relatively larger standard deviation in soybean oil may be attributed to its high market consumption, which increased the risk of uncertain adulteration or blending with other oils, leading to greater variability in fatty acid composition across different brands. Sesame oil, sunflower oil and peanut oil exhibited relatively consistent median palmitic acid contents, among which sesame oil showed the best consistency (mean = 11.5, SD = 1.14) with an outlier. Peanut oil displayed the largest variability (mean = 11.18, SD = 1.69). In contrast, rapeseed oil from all brands had a notably lower palmitic acid content (mean = 4.93, SD = 0.57). Overall, sesame oil, sunflower oil, peanut oil, and olive oil each had one statistical outlier. This indicated that the GC-MS measurement results of palmitic acid content in edible oils had the basic statistical consistency.

### 3.3. Selection of Informative Wavelengths for Palmitic Acid Prediction

**Figure 9:**
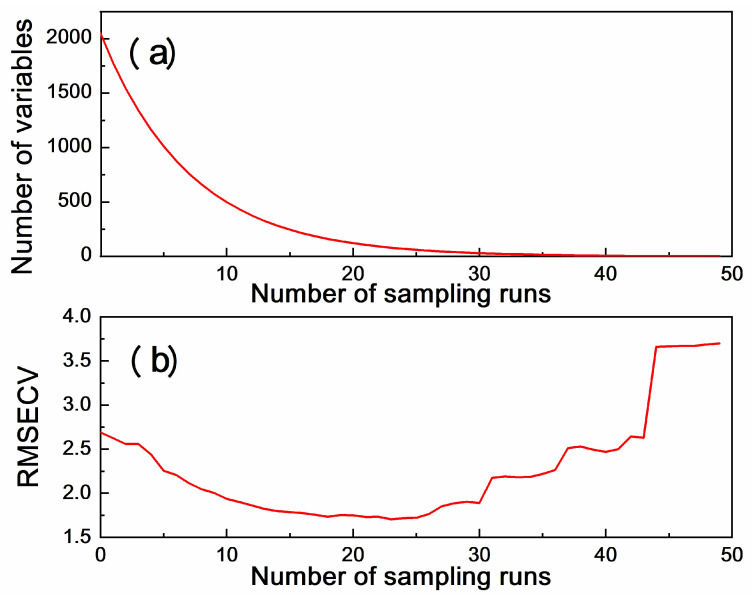
The process of wavelength selection by CARS. (**a**) The number of kept variables as the number of sampling runs, (**b**) The RMSECV (Root Mean Square Error of Cross-Validation) changes as the number of sampling runs.

CARS was employed to select informative wavelengths for palmitic acid prediction by progressively removing uninformative and redundant spectral variables. Through repeated Monte Carlo sampling and adaptive reweighted regression, CARS was able to identify wavelength subsets that were most relevant to the target variable. The wavelength selection process based on 50 Monte Carlo sampling runs is presented in Figure 9. It should be noted that the wavelength selection using CARS was performed on the calibration set, which consisted of 1160 spectra from 58 brands.

**Figure 10:**
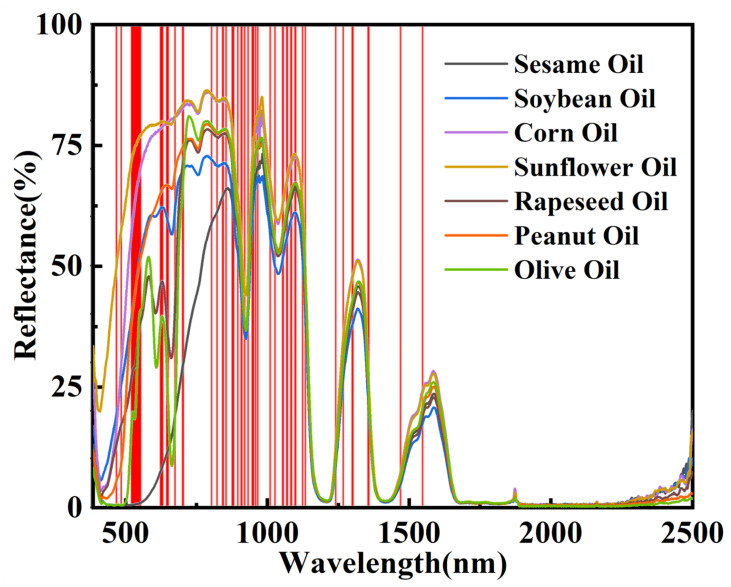
The characteristic wavelengths selected via CARS for quantitative analysis of palmitic acid. The red lines indicated the characteristic wavelengths selected using CARS.

As shown in Figure 9, the number of retained wavelengths gradually decreased as the number of sampling runs increased, whereas the RMSECV initially decreased and then increased. This pattern suggested that many irrelevant or weakly informative wavelengths were removed during the early stage of the CARS procedure. When the number of sampling runs reached 23, the RMSECV attained its minimum value, indicating that the corresponding wavelength subset was optimal. In each iteration, the number of latent variables (LVs) was determined adaptively via cross-validation based on the sample subset and selected wavelengths to minimize RMSECV while capturing essential variance without overfitting. In our experiment, a total of 91 characteristic wavelengths were selected by CARS as being correlated with the measurement of palmitic acid. The selected characteristic wavelengths were listed in Table 1 and shown in Figure 10. These wavelengths were mainly distributed within the range of 470–1550 nm and were concentrated around spectral peaks and valleys, covering characteristic absorption regions associated with molecular vibrations of functional groups such as C-H, O-H, and N-H [24]. This result supported the effectiveness and rationality of CARS for informative wavelength selection. After CARS selection, the number of spectral variables was reduced from 2151 to 91, thereby substantially decreasing data dimensionality.

### 3.4. Full-Spectrum Models for Palmitic Acid Prediction

A systematic comparison of six models was conducted to identify the most suitable approach for the prediction of palmitic acid content based on the full Vis-NIR spectrum. In our experiments, SVR and KNN models were implemented in MATLAB R2016a, whereas 1D-CNN, 1D-ResNet, 1D-Inception, and 1D-Inception-ResNet models were developed in Python 3.10 and pytorch 2.0. All experiments were conducted on a workstation equipped with an AMD Ryzen 5 PRO 2600 six-core processor (3.40 GHz), an NVIDIA RTX 3090 GPU with 24 GB of memory, and 32 GB of DDR4 RAM.

**Figure 11:**
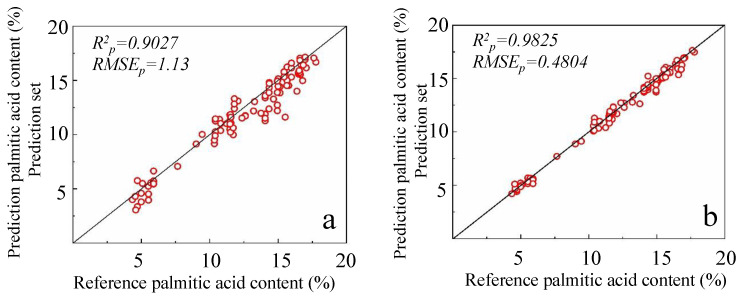
(**a**) Prediction results of the full spectrum based on the 1D-Resnet model and (**b**) prediction results of characteristic wavelengths based on the 1D-Inception-Resnet model.

The experiment results are summarized in Table 2. Overall, the two conventional machine learning models (SVR and KNN) achieved relatively good fitting performance on the calibration set ($R_{c}^{2}$ > 0.91). However, the predictive performance on the prediction set was relatively poor, indicating limited robustness and generalization ability for the prediction of palmitic acid. In contrast, the deep learning models exhibited better predictive performance than the SVR and KNN models. This improvement was likely due to the ability of convolutional neural networks to automatically learn hierarchical and representative features from complex spectral signals. The four proposed deep learning models showed higher predictive accuracy, demonstrating the advantage of deep feature extraction for quantitative analysis of palmitic acid content. Among all of the tested models, the 1D-ResNet model achieved the best overall performance, with $R_{c}^{2}=0.9788$, $RMSE_{c}=0.5577$ and $R_{p}^{2}=0.9027$, $RMSE_{p}=1.13$. These results indicated that the 1D-ResNet model was able to effectively capture the relationship between spectral information and palmitic acid content, thereby providing the strongest predictive ability among the evaluated models. The prediction results of the optimal model are shown in Figure 11a, where most predicted values were distributed close to the reference values, although a few samples still showed noticeable deviations. It is worth noting that the 1D-Inception-ResNet model achieved the highest calibration accuracy ($R_{c}^{2}=0.9988$, $RMSE_{c}=0.1215$), but its performance on the prediction set ($R_{p}^{2}=0.8935$, $RMSE_{p}=1.187$) was inferior to that of 1D-ResNet. This discrepancy suggested that the 1D-Inception-ResNet model may have been more prone to overfitting. Similarly, although the 1D-CNN model also achieved strong prediction performance ($R_{p}^{2}=0.8941$, $RMSE_{p}=1.1843$), it was still slightly less effective than 1D-ResNet. These findings indicated that, for full-spectrum models of palmitic acid in edible oils, the 1D-ResNet architecture provided the best balance between fitting accuracy and generalization ability.

### 3.5. Prediction of Palmitic Acid Content Using Selected Wavelengths

The 91 selected informative wavelengths were subsequently used as input variables to train models for palmitic acid prediction. The corresponding results are summarized in Table 3. Overall, compared with the full-spectrum models, the models developed using the selected wavelengths achieved better predictive performance, indicating that wavelength selection effectively removed irrelevant and redundant spectral variables and retained the most informative features for palmitic acid prediction. Among the two conventional machine learning models, SVR showed moderate predictive ability, with $R_{c}^{2}=0.7897$, $RMSE_{c}=1.7588$ and $R_{p}^{2}=0.7800$, $RMSE_{p}=1.7072$. KNN achieved a perfect fit on the calibration set ($R_{c}^{2}=1.0000$, $RMSE_{c}=0$), but its predictive performance on the prediction set was substantially lower ($R_{p}^{2}=0.7616$, $RMSE_{p}=1.7777$), indicating severe overfitting and limited generalization ability. These results suggested that conventional machine learning models were less effective in capturing the complex nonlinear relationship between the selected spectral variables and palmitic acid content.

In contrast, the four proposed deep learning models exhibited markedly better performance than the machine learning models. The 1D-CNN model achieved $R_{c}^{2}=0.9584$ and $R_{p}^{2}=0.9410$, with corresponding $RMSE_{c}$ and $RMSE_{p}$ values of 0.7816 and 0.8839, respectively, indicating that convolution-based feature extraction could effectively improve predictive accuracy. Compared with 1D-CNN, the 1D-ResNet model further improved prediction performance, yielding $R_{c}^{2}=0.9752$, $RMSE_{c}=0.6036$ and $R_{p}^{2}=0.9528$, $RMSE_{p}=0.7905$. This improvement suggested that residual connections facilitated the learning of more representative spectral features and enhanced model robustness. The 1D-Inception model achieved even better results, with $R_{c}^{2}=0.9845$, $RMSE_{c}=0.4772$, and $R_{p}^{2}=0.9715$, $RMSE_{p}=0.6139$. This result indicated that the multi-branch architecture of the Inception module was effective in extracting spectral information at different receptive field scales. Among all tested models, the 1D-Inception-ResNet model produced the best overall performance, with $R_{c}^{2}=0.9923$, $RMSE_{c}=0.3363$ and $R_{p}^{2}=0.9825$, $RMSE_{p}=0.4804$. The superior performance of this model suggested that combining multi-scale feature extraction with residual learning enabled more effective modeling of the relationship between the selected wavelengths and palmitic acid content.

Notably, the performance of the 1D-Inception-ResNet model based on the selected wavelengths was clearly better than that of the best full-spectrum model. In the full-spectrum analysis, the best 1D-ResNet model achieved $R_{p}^{2}=0.9027$ and $RMSE_{p}=1.13$, whereas the selected wavelength 1D-Inception-ResNet model improved these values to $R_{p}^{2}=0.9825$ and $RMSE_{p}=0.4804$. This result demonstrated that CARS-based wavelength selection not only reduced the number of inputs, but also enhanced model accuracy and generalization ability. The prediction results of the 1D-Inception-ResNet model based on the selected wavelengths are shown in Figure 11b. Compared with Figure 11a, the predicted values were distributed more closely around the reference values, with no obvious large deviations. This finding further confirmed that the combination of informative wavelength selection and deep learning modeling was effective for accurate prediction of palmitic acid content in edible oils.

## 4. Discussion and Conclusions

This study developed a rapid and non-destructive strategy for predicting palmitic acid content in edible oils by combining Vis-NIR reflectance spectroscopy with machine learning and deep learning models. The spectral differences observed among the seven edible oil types indicated that reflectance spectroscopy contained useful chemical information related to compositional variation. Two conventional machine learning models (SVR and KNN) and four deep-learning models proposed in this study (1D-CNN, 1D-ResNet, 1D-Inception, and 1D-Inception-ResNet) were developed and compared for palmitic acid content prediction using both full-spectrum data and selected informative wavelengths. Among the full-spectrum models, the 1D-ResNet model achieved the best overall performance, with $R_{c}^{2}=0.9788$, $RMSE_{c}=0.5577$ and $R_{p}^{2}=0.9027$, $RMSE_{p}=1.13$. After CARS-based wavelength selection, the number of spectral variables was reduced from 2151 to 91, and the 1D-Inception-ResNet model yielded the best prediction results, with $R_{c}^{2}=0.9923$, $RMSE_{c}=0.3363$ and $R_{p}^{2}=0.9825$, $RMSE_{p}=0.4804$. The 1D-Inception-ResNet model achieved superior prediction accuracy compared to SVR and KNN, but at a higher computational cost. In our experiments, due to the small dataset size (87 samples), this lightweight deep network converged within 23s on a standard GPU, while SVR and KNN trained almost instantly on CPUs. The improved predictive performance of the 1D-Inception-ResNet justified the additional computational cost. Therefore, these results indicated that informative wavelength selection not only reduced data dimensionality and computational cost, but also improved model accuracy and generalization ability.

However, the variability of palmitic acid content among the 87 edible oil brands should be carefully considered and discussed. The variability of palmitic acid content can influence the interpretation of model performance metrics such as RMSE and $R^{2}$. Oils exhibiting higher heterogeneity, such as soybean and peanut oils, contributed more to the RMSE due to greater sample-to-sample variation, whereas oils with more consistent palmitic acid levels, such as sesame and rapeseed oils, allowed for more accurate predictions and higher $R^{2}$ values. These observations indicated that the reported RMSE and $R^{2}$ not only reflect the predictive capability of the models but also capture the inherent variability of the target variable across different oil types and brands, highlighting the importance of considering sample heterogeneity when evaluating model performance.

Overall, the combination of reflectance spectroscopy, informative wavelength selection, and deep learning provided an effective approach for quantitative prediction of palmitic acid content in edible oils. This strategy showed promising potential for rapid quality evaluation of edible oils and may be extended to the analysis of other quality-related parameters and other liquid food systems. It should be noted, however, that all samples in this study were collected from mainland China (local supermarkets an and online market, JD.com), which may not fully represent oils from other geographic regions or commercial contexts. Future studies should further validate the robustness and generalizability of the proposed method using more diverse sample sources and external datasets.

## Figures and Tables

**Table 1:** The characteristic wavelengths selected using CARS correlated with palmitic acid.

Methods	Wavelengths (nm)
CARS	471, 472, 487, 488, 525, 530, 531, 532, 533, 534
	535, 536, 537, 538, 542, 543, 544, 545, 546, 547
	548, 549, 550, 551, 552, 555, 556, 627, 628, 629
	630, 631, 632, 633, 634, 648, 649, 650, 651, 652
	677, 705, 706, 807, 826, 845, 847, 858, 879, 885
	899, 911, 912, 913, 922, 935, 936, 951, 954, 962 970
	1013, 1014, 1030, 1031, 1058, 1059, 1060, 1072, 1073, 1074
	1087, 1088, 1089, 1101, 1102, 1126, 1137, 1138, 1243, 1270
	1271, 1272, 1302, 1303, 1304, 1359, 1360, 1472, 1550, 1551

**Table 2:** Prediction results of palmitic acid content in edible oils by different models using full wavelength of spectra.

Methods	$R_{c}^{2}$	${\mathrm{RMSE}}_{c}$	$R_{p}^{2}$	${\mathrm{RMSE}}_{p}$
SVR	0.9176	1.1000	0.7537	1.8064
KNN	0.9166	1.1074	0.6353	2.1983
1D-CNN	0.9832	0.4963	0.8941	1.1843
1D-ResNet	0.9788	0.5577	0.9027	1.1300
1D-Inception	0.9616	0.7500	0.8580	1.3700
1D-Inception-ResNet	0.9988	0.1215	0.8935	1.1870

**Table 3:** Prediction results of the important wavelengths regression model.

Methods	$R_{c}^{2}$	${\mathrm{RMSE}}_{c}$	$R_{p}^{2}$	${\mathrm{RMSE}}_{p}$
SVR	0.7897	1.7588	0.78	1.7072
KNN	1	0	0.7616	1.7777
1D-CNN	0.9584	0.7816	0.941	0.8839
1D-Resnet	0.9752	0.6036	0.9528	0.7905
1D-Inception	0.9845	0.4772	0.9715	0.6139
1D-Inception-Resnet	0.9923	0.3363	0.9825	0.4804

## Data Availability

The data presented in this study are available upon request from the corresponding author.

## Abbreviations

The following abbreviations are used in this manuscript:

Vis-NIR	Visible-near-infrared
GC-MS	Gas chromatography–mass spectrometry
SVR	Support vector regression
KNN	K-nearest neighbor
CNN	Convolutional Neural Network
ResNet	Residual Network
CARS	Competitive Adaptive Reweighted Sampling
